# Physiological effects of *in ovo* delivery of bioactive substances in broiler chickens

**DOI:** 10.3389/fvets.2023.1124007

**Published:** 2023-03-16

**Authors:** Kouassi R. Kpodo, Monika Proszkowiec-Weglarz

**Affiliations:** Animal Biosciences and Biotechnology Laboratory, United States Department of Agriculture, Agricultural Research Service, Beltsville, MD, United States

**Keywords:** *in ovo*, nutrition, growth, microbiota, gastrointestinal tract, chicken

## Abstract

The poultry industry has improved genetics, nutrition, and management practices, resulting in fast-growing chickens; however, disturbances during embryonic development may affect the entire production cycle and cause irreversible losses to broiler chicken producers. The most crucial time in the chicks' development appears to be the perinatal period, which encompasses the last few days of pre-hatch and the first few days of post-hatch. During this critical period, intestinal development occurs rapidly, and the chicks undergo a metabolic and physiological shift from the utilization of egg nutrients to exogenous feed. However, the nutrient reserve of the egg yolk may not be enough to sustain the late stage of embryonic development and provide energy for the hatching process. In addition, modern hatchery practices cause a delay in access to feed immediately post-hatch, and this can potentially affect the intestinal microbiome, health, development, and growth of the chickens. Development of the *in ovo* technology allowing for the delivery of bioactive substances into chicken embryos during their development represents a way to accommodate the perinatal period, late embryo development, and post-hatch growth. Many bioactive substances have been delivered through the *in ovo* technology, including carbohydrates, amino acids, hormones, prebiotics, probiotics and synbiotics, antibodies, immunostimulants, minerals, and microorganisms with a variety of physiological effects. In this review, we focused on the physiological effects of the *in ovo* delivery of these substances, including their effects on embryo development, gastrointestinal tract function and health, nutrient digestion, immune system development and function, bone development, overall growth performance, muscle development and meat quality, gastrointestinal tract microbiota development, heat stress response, pathogens exclusion, and birds metabolism, as well as transcriptome and proteome. We believe that this method is widely underestimated and underused by the poultry industry.

## Introduction

Over the years, the poultry industry has improved genetics, nutrition, and management practices, resulting in fast-growing chickens that reach market weight faster than broilers raised decades ago (1). However, disturbances during embryonic development may affect the entire production cycle and cause irreversible losses to broiler chicken producers. Embryonic development accounts for more than 33% of the entire life of commercial broilers (2). Moreover, the most crucial time in the chicks' development appears to be the perinatal period, which encompasses the last few days of pre-hatch and the first few days of post-hatch (3). During this critical period, intestinal development occurs rapidly (4), and the chicks undergo metabolic and physiological shifts from the utilization of egg nutrients to exogenous feed (4, 5). The nutrients in the egg yolk may not be enough to sustain the late stage of embryonic development and provide energy for the hatching process. In addition, modern hatchery practices cause a delay in access to feed immediately post-hatch, and this can potentially affect the intestinal microbiome, health, development, and growth of the chickens (6–15). One of the technological advances that could help remedy the issue is *in ovo* delivery of bioactive substances. The biggest benefit of *in ovo* administration of bioactive substances during embryonic development is their long-term effects on avian physiology (16).

**Figure 1:**
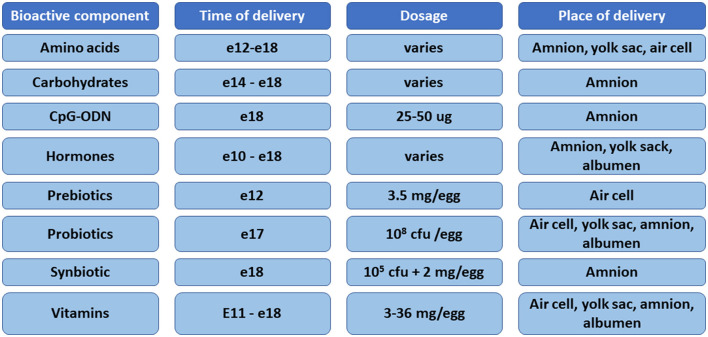
Summary of the bioactive compounds, time of delivery, dose, and sites of injection used for *in ovo* delivery in chickens.

The *in ovo* methodology was developed in the early 1980s as a means of delivering Marek's disease vaccine (17). The development of this technology allows for the expansion of research beyond vaccine delivery. However, many issues have been indicated with the methodology, such as the lack of standardization/optimization of the method for delivery of various bioactive substances, including the age of the embryo, volume of the injection, site of the injection, or concentration of the bioactive substances (3). Automated delivery of bioactive substances has been developed over the years after the initial introduction of the method. The patent of Uni and Ferket (18) is the most widely used method for delivering *in ovo* injections. There are five locations for *in ovo* delivery in chickens, namely, the air cell, embryo, yolk sac, allantoic membrane, and amniotic fluid (3). The *in ovo* technology was adapted over time for *in ovo* feeding of probiotics or prebiotics on embryonic day (e)18 or synbiotics delivery on e12 (18–20). In general, *in ovo* delivery on e12 is referred to as *in ovo* stimulation, whereas delivery on e17–18 is known as *in ovo* feeding (21). According to the patent of Uni and Ferket (18), the optimal time and site for *in ovo* delivery for feeding purposes is late-term embryo and the amniotic fluid, respectively (20). When the embryo consumes the amniotic fluid, intestinal tissues are exposed to the injected substances (18). The *in ovo* procedure on e12 involves delivery of bioactive compounds into the egg's air cell. Prebiotics delivered to the air cell are transferred through the vascular system in the chorioallantoic membrane into the intestine, while probiotics in the air cell are available for the chicken embryo at the time of hatch (19). There is no consensus about the volume of injections. The volume used previously ranges from 50 μl (Marek's disease vaccine), 200 μl (prebiotics and synbiotics), 700 μl (carbohydrates), to 2,000 μl (electrolytes) (22–25). Many bioactive compounds have been studied for *in ovo* delivery, including vitamins, amino acids, carbohydrates, prebiotics, probiotics, synbiotics, hormones, minerals, CpG oligodeoxynucleotides, proteins, and immunostimulants (3, 21) (Figure 1).

**Figure 2:**
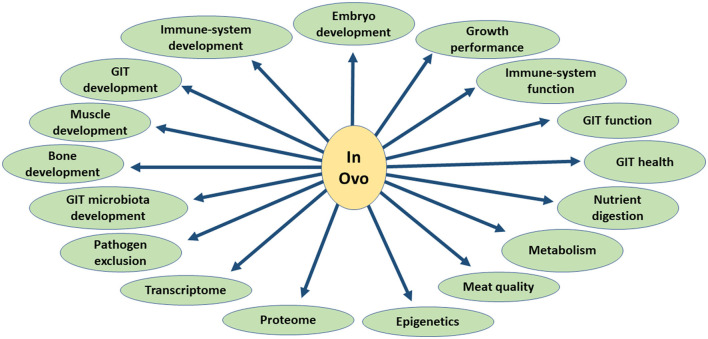
Overview of the biological effects of *in ovo* delivery of bioactive substances.

In this review, we focused on the physiological effects of the *in ovo* delivery of these substances, including their effects on embryo development, gastrointestinal tract (GIT) function and health, nutrient digestion, immune system development and function, bone development, overall growth performance, muscle development and meat quality, heat stress response, GIT microbiota development, pathogens exclusion, and birds metabolism, as well as transcriptome and proteome (Figure 2). The aim of this study was to characterize the physiological changes caused by the *in ovo* delivery of bioactive substances and pinpoint directions where this technology could be used more efficiently to improve the overall growth and health of chickens.

## Embryo development and hatching

Chicken embryonic development is divided into early, middle, and late phases. Organs and systems form during the early and middle phases, and they grow and mature during the late phase. The staging of embryonic development and hatching behavior have been reviewed (26). A successful embryonic development depends on the nutrient reserves of the egg and external factors that create the optimal environmental conditions for the embryo. The metabolism of fast-growing strains of chickens has created the need to supply the developing embryo with nutrients (27), and this has been done throughout the three embryonic phases depending on the objectives but most frequently at the late embryonic phase to increase nutrient reserves before hatch to support the hatching process and early growth post-hatch.

The *in ovo* supplementation targeting the early phase has showed that nanoparticles were able to cross the inner membrane and deliver glutamine into the developing embryo. In this study, diamond nanoparticles in combination with 0.3 ml of L-glutamine (50 mg/L) were injected on e1 in the air chamber. The authors showed that this technology may be able to influence energy metabolism because oxygen consumption was increased on e10, and the proliferation and differentiation of the pectoral muscle cells were enhanced (28).

The supplementation of a mixture of essential and non-essential amino acids solution in the yolk on e7 increased the amino acid contents of the embryo, yolk, albumen, allantois, and amniotic fluid (29). The supplementation during early embryonic development directly impacts organ and system formation; however, the supplementation during late embryonic development increases the energy reserve that will support the hatching process. The hatching process is a coordinated event in which the pipping muscle plays an important role (30). Glucose and glycogen reserves, as well as the weight of the pipping muscle, generally increase during the last 2 days of incubation (31). Uni et al. (32) have demonstrated that the supplementation of a solution containing maltose, sucrose, dextrin, and β-hydroxy-β-methylbutyrate (HMB) during e17.5 increased the energy reserve of the embryo that could be used in the hatching process.

Embryo development can be hindered by the presence of mycotoxins in fertile eggs. Mycotoxins contained in breeders' feed may contaminate eggs (33); cause oxidative stress; and damage organs, tissues, and skeletal muscle in the developing embryo (34, 35). Studies have evaluated the effects of bioactive substances against mycotoxicosis during embryonic development. The supplementation of 0.01 g of *Arctostaphylos uv-aurs* extract (containing phenols and flavonoids) in the amnion on e10 reduced the negative effects of aflatoxin B1 on tibia length and weight (36). Methionine supplementation at 5.90 mg reduced hepatic cell apoptosis caused by aflatoxin B1 toxicity in chicken embryos. However, methionine may have enhanced the oxidative stress caused by aflatoxin B1 as shown by increased superoxide dismutase (SOD), glutathione, glutathione peroxidase (GSH-Px), and catalase activities, despite the reduction in serum malondialdehyde (37). Further research is needed to clarify the effects of methionine and other bioactive substances on mycotoxicosis during incubation in chickens.

## Gastrointestinal tract development

The intestinal development during embryonic stages and early post-hatch depends on the availability of nutrients in the egg. The GIT appears early in embryonic development and follows a sequential formation of its components, but it is completely formed by e12 (38). Morphological and physiological changes start by e15 and increase dramatically when the embryo starts swallowing the amniotic fluid on e17 (39). As a result, the proportion of intestinal to embryo weight increased from 1% on e17 to 3.5% at hatch (39). Physiological functions also appear early and continue to develop until the eggs hatch. Enzyme amylase activity appears on e6 and increases in stages until after e19 (40). Intestinal brush border membranes, or microvilli, increase rapidly in the last 2 days of incubation, thereby expanding the intestinal surface area and increasing the digestive and absorptive capacities (41). Brush border enzymes (maltase and aminopeptidase) and nutrients transporter sodium-glucose cotransporter (SGLT)-1 as well as the basolateral Na+K+ATPase activities increase progressively from e15 and reach their highest levels at hatch (39). Therefore, the intestine of the developing embryo has some but limited capacity to digest carbohydrates and proteins (39).

Embryonic development requires nutrients that are supplied by different substrates through metabolic pathways that are divided into three phases. Gastrointestinal development and metabolic pathways have been previously reviewed (2, 30, 42). Briefly, the embryonic development goes through the germ establishment, the embryonic completion, and the emergence phases. The germ establishment is characterized by anaerobic glycolysis. The embryonic completion is characterized by beta oxidation and the use of fatty acids. During the emergence phase, the embryo switches to glycogen reserves as an energy substrate. The glycogen reserve will be used by the embryo for the hatching process, maintenance, and tissue growth prior to hatch and during the post-hatch period. However, the energy reserve may be depleted, and this can hinder organ development post-hatch. As chicks transition from yolk nutrients to exogenous feed, intestinal development and maturation become crucial factors for survival and growth post-hatch (2, 30, 42). Nutritional strategies during embryonic development are used to improve energy reserves in the *in ovo* technology.

The *in ovo* supplementation of bioactive substances can be used to provide developing embryos with nutrients to improve intestinal development and function during the peri-hatch period. Substances including carbohydrates, amino acids, vitamins, minerals, prebiotics, probiotics, synbiotics, and plant extracts have been investigated and shown to have beneficial effects on intestinal development and digestive function. The supplementation of amino acids can improve intestinal morphology. A 3.5% threonine (0.6 ml) (43, 44) and 6 or 10 mg arginine (45–47) supplemented into the amniotic fluid on e17.5 increased villus height, villus height to crypt depth ratio, and surface area. The supplementation of a mixture of 10 amino acids, providing 16 g/L of total nitrogen in the yolk sac on e14, increased villus height, muscle layer thickness, and goblet density (48). Glutamine supplemented at 1% in the amniotic fluid on e17 increased total villus cell counts during the peri-hatch period (49). Glutamine serves as a fuel source for enterocytes, and this result confirms that it plays an important role in intestinal development.

Carbohydrates such as 10% and 20% dextrose, 0.1% mannan-oligosaccharide (MOS), 0.528 mg transgalacto-oligosaccharide (Bi_2_tos), and 4.5 mg raffinose increased villus height on either e12 or e17 (48, 50, 51). The supplementation of 0.1% MOS, 10% and 20% dextrose, 5% and 10% stachyose, and 1.76 mg inulin on either e12 or e17 increased villus surface area (48, 51, 52). In addition, 0.1% MOS increased the intestinal thickness (50), and 4.5 mg raffinose increased the villus height to crypt depth ratio (51). The supplementation of inulin reduced the crypt depth (53). Different synbiotics of 0.528 mg Bi_2_tos with 1,000 cfu of *Lactobacillus lactis* subsp. *lactis* IBBSL1; 1.76 mg inulin with 1,000 cfu *Lactobacillus lactis* subsp. *cremoris* IBB SC1 (53); and 0.5% inulin with 10^6^ cfu *Enterococcus feacium* (54) increased the villus height.

Villus height, villus height to crypt depth ratio, goblet cell number, and density are morphological markers of intestinal health, and the supplementation of various bioactive substances can increase these markers, suggesting that these substances can improve intestinal development during the peri-hatch period. The significance of these markers generally reported in the literature is based on different sections of the intestine (duodenum, jejunum, and ileum) and different periods of incubation or post-hatch, and this makes the comparison between studies difficult to ascertain the effects of these bioactive substances on the intestine as a whole.

## Immune system development

The immune system development of chickens starts early in the embryonic stage and progresses until hatching. The ontogeny of the immune system in chickens has been extensively reviewed (55, 56). The components of the gut-associated lymphoid tissue develop progressively during embryonic development, as evidenced by the presence of anlage of Peyer's patches and cecal tonsils as well as clusters of MHC class II^+^, IgM^+^, and Bu-1^+^ cells on e15 (57). Toll-like receptors were highly expressed in the heart, brain, intestine, and liver from e12 to e21 (58). Most of the development of the immune system is complete by the late embryonic phase (59); however, the maturation and response of the immune system to antigens increase with age post-hatch (60, 61). Providing substrates and antigens early in life can accelerate the development and maturation of the immune system of chickens. A previous study has shown that early feeding improves the immune system (62); however, due to delayed access to feed, supplying substances prior to hatch is more attractive.

The immunomodulatory effects of prebiotics and synbiotics supplemented *in ovo* have been reported. Prebiotic Bi_2_tos supplemented on e12 in the air sac increased cytokine levels (IL-1β, IL-10, and IL-12p40) in the jejunum, ileum, and cecum as well as avian beta defensin-1 and cathelicidin 2 in the cecum (63). Similarly, a synbiotic (1.9 mg raffinose with 3 × 10^8^ cfu *Lactobacillus lactis* subsp. *lactis* IBB SL1) increased IL-4, IL-6, IFN-β, and IL-18 in the spleen of chickens (64). Not all immune-related genes are upregulated by the *in ovo* supplementation of bioactive substances. Prebiotic (1.76 mg inulin) and synbiotic (1.76 mg inulin with 1,000 cfu of *Lactobacillus lactis* subsp. *lactis* 2955) decreased IFN-β, IFN-γ, IL18, IL-4, IL-6, IL-12p40, IL-8, and CD80 during the post-hatch period, with greater magnitude toward day 35 post-hatch (65). For the authors of this study, the fast-growing strains may have prioritized energy for growth as they reached market weight. However, further studies including some disease challenges after *in ovo* supplementation may help shed light on the effects of these substances on the immune system.

Cell-mediated immunity can also be stimulated by the *in ovo* supplementation of bioactive substances. In a study where prebiotic (3.5 mg Bi^2^tos), probiotic (1 × 10^5^ cfu *Lactococcus lactis* subsp. *cremoris* IBB477), and synbiotic (3.5 mg Bi^2^tos with 1 × 10^5^ cfu *Lactococcus lactis* subsp. *cremoris* IBB477) were supplemented, the probiotic increased the number of CD4^+^ lymphocytes in the cecal tonsils on day 7 post-hatch while the prebiotic increased the number of CD4^+^ and CD8^+^ cells in the cecal tonsils and CD8^+^ cells in the spleen on day 21 post-hatch (66). Other synbiotics (10^3^ cfu *Lactococcus lactis* subsp. *cremoris* IBBSL1 with 1.76 mg inulin or 0.528 mg Bi_2_tos) also increased TCRγδ+ lymphocytes in the spleen at days 21 and 35 post-hatch (67). The supplementation of minerals has been shown to enhance the cellular immune response. Minerals (500 μg Zn, 17.5 μg I, or 1.5 μg Se) supplemented on e14 in the yolk or amnion showed that IL-2 and IL-12 genes expression were increased by Zn, while that of inducible nitric oxide synthase (iNOS) was increased by Zn, Se, and I at day 14 post-hatch (68).

Other immune system parameters such as white blood cell counts, heterophil and lymphocyte ratios, and leucocyte phagocytic ability were mostly increased at day 21 post-hatch by the supplementation of inulin, Bi_2_tos, or synbiotics (inulin with *Lactococcus lactis* subsp. *lactis* or Bi_2_tos with *Lactococcus lactis* subsp. *cremoris*) (69). Other substances such as arginine increased total nitric oxide and iNOS at day 7 post-hatch (45), while vitamin D3 and vitamin K3 increased antibody production (70). The supplementation of soybean selenium proteinate and sodium selenium at e18 in the amniotic fluid increased the IL-6, IL-8, and iNOS genes expression in chickens that were co-infected with *E. maxima* (day 14 post-hatch) and *C. perfringers* (day 18 post-hatch) (71, 72). These results suggest that some prebiotics, probiotics, amino acids, minerals, and vitamins may improve the immune response.

## Muscle development and meat quality

The skeletal muscle formation during embryonic development may influence muscle development and meat quality post-hatch. In general, skeletal muscle fibers are formed through the fusion of myoblasts, and this process is mostly completed by hatch. The fusion of myoblasts is performed by a membrane activator called myomaker, which is required for the formation of multinucleate fibers (73). In chickens, the myomaker gene is highly expressed from e12 to e15 and decreased to nearly zero at hatch, confirming that muscle fiber types are determined during the embryonic development and do not change during the post-hatch period (74). The fiber number and density contribute to muscle growth and weight during post-hatch (75). In addition, intramuscular fat accumulation during embryonic development increases from e17 to day 1 post-hatch and plays an important role in meat quality. Thus, the *in ovo* programming of muscle development and meat quality post-hatch may be possible.

The supplementation of Bi_2_tos with *Lactobacillus salivarius* (SYN1) or raffinose with *Lactobacillus plantarum* (SYN2) supplemented on e12 in the air chamber showed that SYN1 increased the number and diameter of capillaries and decreased fiber necrosis in the breast meat at day 45 post-hatch. Normal breast meat should have enough blood supply, which is increased by the number of capillaries surrounding muscle fibers. Fewer capillaries reduce blood supply to the fiber and can cause necrosis and affect meat quality (76).

Creatine pyruvate (12 mg) supplementation on e17.5 in the amniotic fluid increased pectoral muscle weight on e19 and days 3, 7, and 19 post-hatch. In addition, muscle glycogen was increased at day 7 post-hatch. Body weight was increased at day 21 post-hatch (77). The increase in glycogen reserve upon entering the hatching process may help improve body weight during the post-hatch period. However, not all bioactive substances have shown positive effects on muscle development or meat quality. In a study where raffinose (1.9 mg) was supplemented in the air chamber on e12, meat quality was negatively affected. In addition, raffinose increased peroxidation in the breast meat after slaughter, and this could negatively affect the quality and shelf life of the meat (78).

The supplementation of *Lactococcus lactis* subsp. *cremoris* (10^5^ cfu/egg), Bi_2_tos (3.5 mg/egg), or Bi_2_tos (3.5 mg/egg) with *Lactococcus lactis* (10^5^ cfu/egg) injected into the air chamber on e12 showed no effects on muscle microstructure (fiber diameter, normal fiber, fiber atrophy, giant fiber, change in fiber shape, necrotic fiber, and fiber splitting) (79). The supplementation of arginine at different concentrations (6.25, 25, and 100 mg/kg) on e16 stimulated myoblast differentiation but reduced the number of myofibers and the subsequent muscle growth during the post-hatch period (80). It appears that the supplementation of arginine has negative effects on muscle development and may not be of interest. However, the supplementation of diamond nanoparticles in combination with 0.3 ml of L-glutamine (50 mg/L) on e1 in the air chamber enhanced the proliferation and differentiation of pectoral muscle cells (28). Unfortunately, the authors did not go beyond the embryonic development stage; therefore, the impact of nanoparticles and L-glutamine on muscle development and growth are only limited to incubation time. The *in ovo* programming of muscle development may depend on the substances and the timing of bioactive substance supplementation during embryonic development.

## Bones

The structural soundness is crucial for fast-growing strains of chickens that have been genetically selected for maximum muscle development because of potential negative effects on bone development during embryo development and the early post-hatch period. The bone development in chicken embryos has been reviewed elsewhere (81, 82). In brief, bone formation begins early in embryonic development (83). The skeleton has different structural elements, whose formation is not completed at the same time. The vertebral body formation begins early in embryonic development and continues with differentiation and mineralization until e19 (84). Wing and leg bones are partially mineralized by e12, but the mineralization is mostly completed by e16. For ribs and pelvic bones, mineralization is initiated at e12 (85). Tibia bone elongation and mineralization increase between e14 and e17, while those of the femur occur between e19 and e21 (86). Regardless of the sequence of bone formation and mineralization, minerals are exported from the egg yolk and eggshell at different periods. While the yolk supplies most minerals during early phase of embryo development, eggshell supplies a greater amount of minerals between e12 and e16 (85). Chicken bone formation and mineralization slow down during the late embryonic phase, and this coincides with the depletion of the egg mineral (Cu, Fe, Zn, P, and Mn) reserves (86, 87). In addition, the muscle development of fast-growing strains puts more pressure and load on their skeleton, resulting in all forms of bone deformities and leg problems (88). Therefore, the supplementation of minerals during the late embryonic phase could increase the mineral uptake by the developing embryo and increase mineral reserves to support the rapid bone development and mineralization during the post-hatch period. In addition, vitamin D and vitamin K are two principal actors that regulate the mobilization of minerals from the yolk and eggshell, and their supplementation in combination with minerals during embryonic development may increase mineral absorption, at least Ca and P, and improve bone development (70, 89).

The supplementation of organic trace minerals (P, Zn, Cu, and Mn), phosphate, and vitamin D; inorganic trace minerals (P, Zn, Cu, and Mn) and phosphate (90); and maltodextrin containing Fe, Zn, Mn, Ca, Cu, and P (87) on e17 in the amniotic fluid increased the Zn, Cu, and Mn contents of the yolk on e19 and at hatch. Unfortunately, these studies (87, 90) did not determine mineral content beyond hatch. Contrary to these results, Oliveira et al. (91) reported no effects of *in ovo* supplementation of Mn, Zn, and Cu on mineral (Ca, P, and Zn) content at days 1, 7, and 21 during the post-hatch period. There were no effects of *in ovo* supplementation with 25-hydroxycholecalciferol (vitamin D3) on bone mineral contents (92). These discrepancies in the literature regarding the effects of minerals and vitamin supplementations on bone mineral contents are likely due to the nature of the supplements, the organs used (yolk as opposed to bone), and the periods of sampling. Further research is needed to investigate the effects of the *in ovo* supplementation of minerals and vitamins covering at least the late embryonic phase and the life span of fast-growing strains of broiler chickens.

The main objective for supplying the developing embryo with minerals or vitamins is to reduce mineral deficiency during peri-hatch period and improve bone development and characteristics. The *in ovo* supplementation of minerals (Zn, Cu, Mn, and Ca) and vitamin D3 improved tibia and femur length, stiffness and thickness, load and work to fracture, breaking strength, and bone weight during the post-hatch period (90). Similarly, the supplementation of 0.6 μg of vitamin D3 and vitamin K3 in the amniotic fluid on e18 improved tibia fracture force, bone weight, and breaking strength of the bone while higher doses of vitamin D3 >0.6 μg reduced bone strength (70). Contrary to the studies (87, 90, 91) mentioned in the previous paragraph, others have reported no effects of *in ovo* supplementation of minerals and vitamins on bone characteristics. No treatment effects of supplemental minerals (Zn, Mn, and Cu) on bone Ca, P, Mg, Mn, and Zn concentrations despites increased % ash of the bone (93). In addition, no effects of vitamin D supplementation on breaking strength were reported by Bello et al. (92). It is worth mentioning that the dosage, type, and combination of these minerals with vitamins may explain the differences. Alternatively, higher dosages of minerals and vitamins may incur some toxicity or mineral imbalance that may have confounded the results of these studies.

## Microbiota development

Chicken GIT microbiota is composed of bacteria, fungi, viruses, and protists and is characterized by commensal, symbiotic, and pathogenic relationships with its host (94). Bacterial species identified in the broiler chicken's GIT (95) play important roles in host nutrition, including feed digestion, nutrient absorption and metabolism, pathogen exclusion, endocrine activity, immune system development and functioning, and performance efficiency (96). In broiler chickens, the symbiotic relationships between the host and the microbiota have been characterized by nutrient exchange, modulation of the immune system, GIT physiology, and pathogen exclusion (6, 95, 97–99). Microbiota composition and function can be affected by many factors, including age; host genotype and sex; diet composition and form; dietary ingredients such as probiotics, prebiotics, synbiotics, phytobiotics; and bacteriophages, stress, antibiotics, and location in the GIT (97, 99, 100). Colonization of the newly hatched GIT occurs through a process known as ecological succession (101). The GIT microbiota reaches the mature community state between weeks 2 and 3 post-hatch (102). Early colonization of beneficial bacteria in the GIT can prevent not only intestinal disorders caused by pathogenic bacteria but also promote the maturity and integrity of the GIT (103). Any improvement in early GIT maturation and digestive ability will positively impact the growth and production performance of chickens (50). *In ovo* injection has the possibility to change bacterial composition in GIT short-term and long-term and influence its developmental process. Most of the studies published and summarized below focus on the effects of probiotic delivery on GIT colonization and microbiota balance.

Pedroso et al. (104) showed that *in ovo* administration on e18 of a probiotic competitive exclusion product derived from adult chicken microbiota influenced the development of broilers microbiota in both fast-growing and heritage-breed chickens. They showed that the early administration of microbiota increased the diversity and taxonomic composition of recipient microbiota (104). Additionally, in heritage birds, the abundance of undesirable bacteria phylum such as Proteobacteria was reduced by *in ovo* treatment (104). Injection of *Bacillus subtilus, Entrococcus faecium*, or *Pediococcus acidilactici* (10^7^ cfu) on e18 into the amnion decreased the population of *E*. *coli* and increased the abundance of lactic acid bacteria during the first week post-hatch (105). Supplementation of lactic acid bacteria probiotic, Flora-Max B11 (10^4^ cfu/egg into the amnion), on e18 influenced the microbiota composition in the GIT by increasing the abundance of lactic acid bacteria post-hatch (106). Additionally, *in ovo* delivery of Flora-Max-B11 reduced the salmonella incidence in comparison to non-treated birds (106). Injection of different *Bifidobacteriun* spp. (2 × 10^8^ cfu of *B. bifidum, B*. *animalis, B. longum*, or *B. infantis*) into the yolk sac on e17 increased the abundance of lactic acid bacteria and decreased the total counts of bacteria and coliform in ileum (107). Wilson et al. (108) have shown that supplementation with 10^2^ cfu of lactic acid bacteria such as *Citrobacter freundii* or *Citrobacter* spp. on e18 influenced the initial microbiota of the ceca. Moreover, changes in ileal microbiota diversity were observed after *in ovo* inoculation of *Citrobacter freundii, Citrobacter* spp., or lactic acid bacteria mixture on e18 into the air cell (10^2^ cfu/egg) at days 3 and 10 post-hatch (109). Lactic acid bacteria supplementation increased the colonization of butyrate-producing bacteria and decreased the level of Enterococcaceae (109). Another study has shown that inoculation of embryos with lactic acid bacteria isolated from adult hen on e19 (10^7^ cfu/ml) induced variation in cecal microbiota as shown on day 7 post-hatch. This variation was characterized by a reduction in Enterobacteriaceae, an increase in the abundance of Ruminococcaceae, and an enrichment of *Proteus, Butyricicoccus*, Lachnospiraceae, and Erysipelotrichaceae (110).

Probiotics and prebiotics functions are different in GIT; the prebiotics stimulate the development of microbiota and improve the microbiota balance, whereas probiotics colonize the environment (111, 112). Supplementation with two different synbiotics (10^5^ cfu *L. salivarius* and 2 mg GOS or 10^5^ *L. plantarum* and 2 mg of raffinose-family oligosaccharides per egg) on e12 resulted in changes in microbiota composition in ileum and ceca on day 21 post-hatch as determined by fluorescent *in situ* hybridization (113). Both synbiotics decreased the abundance of *Lactobacillus spp*. and *Enterococcus* spp. in the ileum, while in the cecum, *Bacteroides-Prevotella* cluster was lowered and *Eubacterium rectale* cluster was increased in comparison to non-injected chickens (113). GOS (3.5 mg/egg) supplementation on e12 (air cell) significantly decreased the relative abundance of *Lactobacillus spp*. in the ileum and increased the level of *Bifidobcterium* spp. in the cecum at 42 days post-hatch (63).

Other studies have shown that *in ovo* supplementation with chito-oligosaccharide and chlorella polysaccharide (5 or 20 mg/egg) on e12.5 led to changes in cecal microbiota later in life (day 21 post-hatch) (114). The relative abundance of polysaccharide-utilizing bacteria such as *Lactobacillus johnsonii, Bacteroides coprocola*, and *Bacteroides salanitronis* was higher in supplemented birds. At the same time, the abundance of opportunistic pathogenic bacteria was decreased due to the supplementation (114). Interestingly, the supplementation did not influence the development of microbiota, since no changes in taxonomic composition were observed at 3 days post-hatch (114). Injection of the essential amino acid, i.e., arginine (0.5–1%) into the amniotic fluid on e14 increased the abundance of *Lactobacillus* and decreased the levels of *Coliform* and *E. coli* bacteria (115).

Besides synbiotics, probiotics, and prebiotics, microbiota development has also been shown to be influenced by other bioactive substances, such as host-defense peptides or antioxidants. Cuperus et al. (116) showed that *in ovo* injection of the host-defense peptide—chicken cathelicidin-2 (1 mg/kg of body weight, e18 into the amnion) altered the GIT microbiota by reducing the abundance of Ruminococcaceae and *Butyricicoccus* in the ceca and *Escherichia/Shigella* in the ileum and ceca at 7 days post-hatch. Intraamniotic administration of isoflavone genistein (1.25 and 2.5%) on e17 positively altered the composition and function of the intestinal microbiota by increasing the abundance of *Bifidobacterium* spp., *Lactobacillus* spp., and *Clostridium* spp. and decreasing the level of *E. coli* (117).

## Metabolism

Adequate energy reserve in the embryo of fast-growing chickens is the basis for later successful growth performance. The metabolic pathways in poultry embryos prior to hatch have been reviewed elsewhere (2, 42). During the late embryonic phase, the developing embryo increases its energy reserves through gluconeogenesis and glycogenesis using protein from the amniotic fluid and tissue reserve. Gluconeogenesis occurs mainly in the yolk sac, but also in the liver during late embryonic development (118). The synthesized glycogen is then stored in the liver, muscles, and yolk (118). These energy reserves will be used for the hatching process and during the early post-hatch period for maintenance and development (2, 42, 119). However, the yolk nutrient reserves are depleted during the late embryonic phase, and the muscle of the developing embryo becomes the main source of amino acids for gluconeogenesis. In addition, after hatching, chicks rely on the residual nutrients of the yolk as they transition to exogenous nutrient sources (32). As modern chicken strains have been selected to grow about twice as fast with liver maturing earlier in heritage lines (120), embryonic development has become a crucial phase to prepare and equip newly hatched chicks for this fast-growing process. In the last two decades, studies have focused on supplying various nutrient substrates to the developing embryo, more specifically, during the late embryonic phase between e14 and e18, to increase energy reserve and other nutrients that will support organs and structural development post-hatch.

A mixture of 1 g/L β-hydroxy-β-methylbutyrate, 25 g/L maltose, 25 g/L sucrose, and 200 g/L dextrin supplemented on e17.5 in the amniotic fluid increased liver and pectoral muscle glycogen at e20, hatch, and days 10 and 25 post-hatch (32). Similarly, 15 mg creatine monohydrate in combination with 62.5 mg glucose injected in the amniotic fluid on e17.5 increased muscle glycogen on e19 (121). The supplementation of 12 mg creatine pyruvate injected in the amniotic fluid on e17.5 also increased liver glycogen reserve on e19 and at hatch (77).

Various substances can be used to increase energy reserves; however, it is important that these substances or the end products of their digestion or hydrolysis can serve as substrates for gluconeogenesis. It is possible that the increase in energy reserve has spared muscle catabolisms resulting in increased body weight and organ development at hatch which were sustained during the post-hatch period (32, 77, 121). The *in ovo* injection of nucleosides on e18 (25–100 mg/egg) led to significantly higher metabolizable energy in chickens on day 14 post-hatch (122). Nucleosides, the materials for nucleic acid synthesis, are suggested to play an important role in the development of the GIT and immune system (122). Supplementation of creatine pyruvate (12 mg/egg) on e17.5 increased energy reserves in broilers (123). The same dose of creatine pyruvate increased plasma creatine and pyruvate concentration, liver pyruvate, glycogen and glucose concentration, and mRNA expression of gluconeogenesis and glycogenesis enzymes in the liver before hatch or early post-hatch (124).

Furthermore, *in ovo* stimulation of embryos with *Lactobacillus*-based synbiotics on e12 increased glucose, insulin, glucagon, and leptin levels in plasma and influenced the expression of metabolic genes in muscle (125). However, more changes in metabolic gene expression were seen early post-hatch (day 7) vs. the end of the production cycle (day 42). Among the metabolic genes, follistatin, pyruvate dehydrogenase kinase, isozyme 4, CCAAT/enhancer binding protein beta, phosphorylase kinase regulatory subunit beta, AMP-activated protein kinase, and gamma 3 subunit were significantly upregulated due to synbiotic delivery (125).

## Gastrointestinal health

Intestinal health is a complex and dynamic interaction involving the intestinal mucosa and microbiota, the immune system, and feed utilization. This section will mainly focus on the effects of *in ovo* supplementation with bioactive substances on the intestinal mucosa. The epithelium is the mucosal layer that forms the direct interface between the body and the intestinal lumen. It is formed by a single cell layer made of different cell types, including enterocytes, goblet cells, enteroendocrine cells, Paneth cells, and tuft cells (126). The intestinal barrier formed by the epithelial cells is guarded by a complex structure including the tight junction, adherent junction, gap junction, and desmosome that spans the paracellular space. The most apical component, the tight junction, comprises the transmembrane proteins claudins (CDN), occludin (OCLN), and the scaffolding molecules of the cytoskeleton, zonula occludens (ZO). The tight junction regulates paracellular transport through the intestinal barrier by selectively preventing harmful molecules and pathogens from entering the body while allowing the passage of smaller molecules and nutrients (127). The intestinal mucosa is completely formed at hatch, but morphological and physiological changes continue during the post-hatch period (41). During early post-hatch when chicks transition from using nutrients from the residual yolk to exogenous feed, their immature intestine is more vulnerable to opportunistic pathogens and toxins from the feed (128). Although, early feeding during post-hatch to provide nutrients to hatchlings has been developed, *in ovo* programming of intestinal barrier functions has gained attraction recently.

The *in ovo* supplementation of bioactive substances can provide nutrients not only for energy reserve to support early life but also to improve the development of the intestinal mucosa and its barrier components. In general, tight junction proteins are assessed through the expression levels of their respective genes. Arginine supplementation (6 or 10 mg) increased CDN-1, ZO-1, and ZO-2 mRNA expression levels (47). Despites the crucial role of tight junctions in intestinal health, limited reports exist on the effects of *in ovo* supplementation of bioactive substances on its different components. Further research is warranted to assess the effects of *in ovo* supplementation not only on gene expression of the tight junction proteins but also on the expression of their protein levels in the intestinal mucosa.

In addition to the tight junctions, the intestinal epithelium is covered by a protective mucus layer, which plays a crucial role in the intestinal defense mechanism. The mucus layer is formed by the gel-forming mucins (MUC) produced by the goblet cells. Different types of mucins exist, but the MUC2 gene expression level has been extensively used as a marker of intestinal health in poultry. Because the intestine is still immature at hatch and undergoing morphological and physiological changes (41), the presence of the mucus layer is critical for its protection against pathogens and toxins present in feed. The *in ovo* supplementation of bioactive substances can modulate the gene expression levels of MUC2. The gene expression level of MUC2 was increased by the *in ovo* supplementation of threonine, arginine, and methionine (43, 44, 47, 129). The supplementation of 10^7^ cfu of *B. subtilis* on e18 increased MUC2 gene expression in intestinal mucosa at e21 and day 3 post-hatch (105). Goblet cell numbers and density were increased when methionine, glutamine, and synbiotics (inulin + *Lactobacillus lactis* subsp. *lactis* IBBSL1) were supplemented *in ovo* in chickens (53, 129, 130). Although the MUC2 gene expression has been extensively used in poultry as a marker of intestinal health, gene expression may be transient and may not reflect the actual protein level of the mucin. In addition to the gene expression, the mucus thickness may be of interest since the mucus layer may not cover the entirety of the intestine as reported in rats (131). Further research needs to determine mucin protein levels and mucus thickness to expand our knowledge of *in ovo* supplementation on intestinal mucosa barrier function.

## Digestion

The small intestine plays an important role in the digestion and absorption of nutrients. Although the intestine is completely formed and functional at hatch, it continues to develop and mature as the chick transitions to exogenous nutrient sources (132). The main accessory digestive organ, the pancreas, also continues to develop, as evidenced by its histogenesis during the post-hatch period (133). The activity of digestive enzymes secreted by the pancreas, including amylase, trypsin, lipase, and chymotrypsin, has been shown to increase during the post-hatch period (134). Because the intestine is immature at hatch and fast-growing birds in the modern poultry industry have been genetically selected to reach market weight by 6–8 weeks after hatch, the intestine needs to reach its full functional digestive and absorptive capacities quickly. Luminal digestion can be improved through *in ovo* supplementation of bioactive substances that enhance digestive enzyme activities. In this regard, the *in ovo* supplementation of 1% arginine increased maltase and sucrase activities at day 7 post-hatch (45). Synbiotics, 0.528 mg Bi_2_tos with 1,000 cfu of *Lactobacillus lactis* subsp. *lactis* IBBSL1 and 1.76 mg inulin with 1,000 cfu *Lactobacillus lactis* subsp. *cremoris* IBB SC1, injected into the air sac on e12 enhanced amylase, lipase, and trypsin activities post-hatch (135).

In addition to enzymatic activity in the intestinal lumen, nutrient digestion is completed with other enzymes at the brush border membrane of enterocytes (136). Reicher and Uni (41) recently reported that the development and maturation of the intestinal digestive and absorptive functions depend on intestinal mucosa growth and surface expansion, both of which take more time during the post-hatch period. Structural changes can still occur over 7 days post-hatch when the intestinal mucosa reaches its maximum growth rate (41). This suggests that the newly hatched chicks have limited capacities to complete the digestion and absorption of carbohydrates, di-, and tripeptides in their early days post-hatch. Improving intestinal digestive and absorptive capacities is nowadays possible by increasing brush border membrane enzyme activities and nutrient transporters early during embryonic development (5). The *in ovo* supplementation of 0.1% MOS increased the aminopeptidase and sucrase-isomaltase activities of the brush border membrane (50). Prebiotics (5% and 10% of raffinose and stachyose) upregulated the mRNA expression of aminopeptidase and sucrase-isomaltase genes (52). A 3.5 % threonine *in ovo* supplementation increased mRNA expression of aminopeptidase N gene (43). Furthermore, *in ovo* supplementation of bioactive substances can improve nutrient transporter activities. For example, glutamine increased the differentiated di- and tri-peptide transporter 1 (PepT-1) region on intestinal villi (49). *In ovo* supplementation of threonine increased the mRNA expression of SGLT-1, glucose transporter 2, and alanine, serine, cysteine, threonine (ASCT) transporter 1 (137) and the expression of PepT-1 (43, 137). Prebiotics (5% and 10% of raffinose and stachyose) upregulated the mRNA expression of SGLT-1 (52). Intestinal luminal and mucosal digestive and mucosal absorptive capacities can be improved by *in ovo* supplementation of various bioactive substances.

## Birds' performance

Current broiler lines are characterized by rapid growth, high body weight at slaughter, and a reduced feed conversion ratio. The *in ovo* supplementation of bioactive substances has been shown to influence chicken performance parameters. Body weight at hatch or during the post-hatch period was increased by *in ovo* supplementation of Bi_2_tos (24, 138), *Bacillus*-based probiotics (139); lactic acid bacteria-based probiotics (110); a mixture of *Lactobacillus acidophilus, Lactobacillus casei*, and *Bifidobacterium bifidum* (140); inulin; and *Lactococcus lactis* spp. *lactis* 2955 (24). Furthermore, arginine (47) and 60 mg Zn injected on e18 in the amniotic fluid (141) increased body weight and feed intake and decreased feed conversion ratio (46, 47). Production parameter reports are not always consistent in the literature. Recent reports have highlighted the fact that some bioactive substances have had unintended results by increasing feed intake and feed conversion ratio. Some synbiotics, such as raffinose with *Lactococcus lactis* ssp. *cremoris* IBB SC1, lactose with *Lactobacillus acidophilus* and *Streptococcus faecium* (142), and *Lactobacillus plantarum* with Astragalus polysaccharide, or probiotics, such as *Lactobacillus plantarum* with an increased feed conversion ratio (143). These conflicting results are likely due to the types of bioactive substances, dosages, environmental conditions, and management differences. In addition, the periods considered for feed intake, body weight gain, and feed conversion ratio are not always the same across studies. To advance our knowledge of the impacts of bioactive substances on production parameters, further research needs to take these factors into consideration and develop standards that can facilitate the transfer of results and the adoption of these new *in ovo* supplementation technologies by the poultry industry.

## Epigenetic regulation

Epigenetic modification of gene expression works through the methylation of DNA. This process can be modulated by nutritional and stress factors as well as intestinal microbiota (144–146). Dunislawska at al. (147) showed that *in ovo* delivery of *Lactobacillus*-based synbiotics on e12 significantly changed methylation pattern of several hepatic genes related to metabolism. Similarly, microbiota modulation by prebiotics such as GOS and inulin, or *Lactobacillus*-based synbiotics have shown an ability to modulate the methylation pattern of splenic genes in fast-growing broilers and native chickens (148). However, the epigenetic changes depended on the chicken genotype and the bioactive substances delivered *in ovo* (148). It has been suggested that the mechanisms of negative changes in gene expression in the liver of broiler chickens after *Lactobacillus*-based synbiotic injections are probably related to changes in DNA methylation patterns and miRNA activity (147, 149). Additionally, Zhu et al. (150) have shown that *in ovo* delivery of vitamin C on e11 resulted in changes in the mRNA expression of genes related to DNA methylation such as DNA methyltransferase, DNA demethylation, growth arrest, and DNA-damage inducible protein beta, thymine-DNA glycosylase, and methyl-CpG-binding domain protein 4 at day 21 or 42 post-hatch.

## Transcriptome

*The in ovo* delivery of bioactive compounds has been shown to affect gene expression in several tissues in developing embryos and post-hatch chickens. Injection *in ovo* of prebiotics and synbiotics on e12 influenced the transcriptomic profile of the spleen, cecal tonsils, and large intestine post-hatch (151). The most differentially expressed genes were observed in the cecal tonsils, and galacto-oligosaccharides (GOS) were the most potent stimulators of the host transcriptome (151). Moreover, lymphocyte proliferation, activation, and differentiation, as well as cytokine production, were the most affected gene ontology categories in cecal tonsils (151). Previous studies from the same group have shown that early delivery of synbiotics composed of raffinose family oligosaccharides and *Lactococcus lactis* upregulated and downregulated interleukin mRNA expression in the spleen and cecal tonsils, respectively (64). Downregulation of the expression of immune-related genes in the spleen and the cecal tonsils was observed after injecting the chicken embryos on e12 with inulin or GOS together with *Lactococcus lactis* (65). Synbiotics, *Lactobacillus salivarius* plus GOS and *Lactobacillus plantarum* plus raffinose family oligosaccharides, injected *in ovo* on e12 decreased expression of two incretins: glucagonlike peptide 1 (GLP-1) and glucose-dependent insulinotropic peptide (GIP) mRNA in the duodenum and GLP-1 receptor mRNA in pancreas post-hatch (152). Both incretins are involved in many biological functions (153). The study by Kolodziejski et al. (152) suggests that synbiotics can be directly or indirectly involved in incretin secretion and action. Injection of synbiotics also resulted in transcriptome changes in the spleen, jejunum, cecal tonsils, and liver on day 35 post-hatch (154). These authors showed that the synbiotic based on *Lactobacillus salivarius* was involved in the activation of pathways related to fat and carbohydrate metabolism, cell adhesion, and immune response, while the synbiotic based on *Lactobacillus plantarum* was involved in the upregulation of expression of genes involved in metabolic pathways (154). In another study, Bertocchi et al. (155) showed that *in ovo* supplementation with prebiotics (GOS) did not result in any differentially expressed genes in the jejunum and cecum post-hatch; however, gene set enrichment analysis (GSEA) showed 11 significantly enriched gene sets related to energy metabolism and oxidation in the jejunum. In contrast, very few changes in enriched gene sets were observed in the cecum after *in ovo* injections of GOS (155). The supplementation of GOS on e12 (3.5 mg/egg) attenuated the negative effects of acute and chronic heat stress post-hatch by decreasing the mRNA expression of IL-4, IL-12p40, and SOD in comparison to non-injected birds (156). Additionally, it has been shown that administration of *Lactobacillus*-based synbiotics affects the metabolism and development-related gene expression in the liver in broiler chicken (154), while *in ovo* administration of inulin (1.76 mg/egg) as a prebiotic or inulin and *Lactococcus lactis* (1.76 mg + 1,000 cfu/egg, respectively) on e12 led to changes in immune gene expression in peripheral tissues, cecal tonsils, and spleen (65). Besides synbiotics having wide effects on gene expression in multiple tissues, probiotics alone have been shown to influence mRNA levels. Embryonic injection of multi-strain lactobacilli mixture composed of *Lactobacillus salivarius, L. reuteri, L. crispatus*, and *L. johnsonii* on e18 (10^5^-10^7^ cfu/egg) led to changes in cytokines mRNA expression in cecal tonsils, bursa of Fabricius, and spleen at 5 and 10 days post-hatch (157). Upregulation of cytokines (IFN-α, IFN-β, IFN-γ, IL-8, and IL-12) expression was observed in the spleen, while cecal tonsils were characterized by downregulation of IL-6, IL-2, and IL-8 post-hatch. In the bursa of Fabricius, only IL-13 expression was affected by *Lactobacillus in ovo* delivery (157).

Although several studies utilized synbiotics or probiotics, transcriptome has also been shown to be influenced by the delivery of other bioactive substances. For example, intra-amniotic zinc-methionine administration on e17 increased zinc exporter mRNA expression in the small intestine as well as the expression of brush border enzymes and transporter genes, suggesting its role in intestinal development enhancement and improved functionality (158). Zinc plays an important role in many biological processes, and it is required for enzyme function, nucleic acid synthesis, and as a cofactor in many proteins (159–162).

Other studies have shown that perinatal administration of 2 mM quinine on e17 affected the expression level of gustatory (palate) and extra-gustatory (duodenum) mRNA for the three bitter taste receptors ggTas2r1, ggTas2r2, and ggTas2r7 and their signaling components alpha-gustducin, phospholipase, and transient receptor potential melastatin 5 (163). Amino acids have also been implicated in gene expression regulation. Threonine injection (from 17.5 to 70 mg/ml) into amniotic fluid on e17 resulted in increased mRNA levels of the MUC2 gene and PepT-1 at hatch, suggesting the role of threonine in the functional development of intestinal mucosa (43).

Zhao et al. (123) showed that creatine pyruvate (12 mg/egg) *in ovo* injection on e17.5 increased the expression level of myogenic differentiation 1, myogenin, and paired box 7 at day 3 post-hatch, leading to enhanced muscle growth and increased satellite cells activity (123). Moreover, injection of creatine pyruvate affected the expression level of gluconeogenesis and glycogenesis enzymes in the liver during the late embryonic phase and early post-hatch indicating that *in ovo* delivery of creatine pyruvate may increase energy reserves in the liver in broiler chickens early post-hatch (124).

Few studies have also shown the effects of growth factors and vitamins on transcriptomics. Epidermal growth factor (EGF) has been shown to be involved in the stimulation of proliferation, differentiation, and maturation of neonatal intestinal cells in mammals (164, 165). In birds, *in ovo* delivery of EGF (160–640 μg/egg, e17) increased EGF receptor mRNA expression only during the end of embryonic development and had no effects post-hatch (166). Supplementation of vitamin C (3 mg/egg into amnion on e11) regulated the expression of inflammatory cytokines while the same dose injected on e18 into the air sac decreased the expression of proinflammatory cytokines such as IL-1β, IL-6, and TNF-α in the spleen and increased expression of antioxidant genes such as GSH-Px and SOD (150, 167).

## Proteome

Information addressing the changes in proteome due to *in ovo* delivery is limited. Dunislawska et al. (168) have shown that synbiotics based on *Lactobacillus plantarum* and raffinose family oligosaccharides administered *in ovo* on e12 led to changes in the expression of the liver proteome in chicken post-hatch. Injection of 200 μl of a synbiotic, 2 mg/egg of oligosaccharides, and 10^5^ cfu of *Lactobacillus plantarum* into the air chamber resulted in 16 differentially expressed proteins (5 downregulated and 11 upregulated) in chicken liver at 21 days post-hatch. Analysis of the differentially expressed proteins determined that they belong to mitochondrial, cytoskeleton, cytoplasmic, and cytoskeleton organizing proteins (168). The authors concluded that *Lactobacillus*-based synbiotics have the ability to accelerate the energy-yielding metabolic pathway in the liver of 21-day-old broilers (168).

Wilson et al. (169) have found that *in ovo* administration of 10^2^ cfu (200 μl volume, into amnion) of *Citrobacter freundii, Citrobacter* spp., or mixed of *Lactobacillus salivarius* and *Pediococcus* spp. on e18 resulted in the changes in 107, 39, and 78 proteins level in the gastrointestinal tract at hatch. These proteomic changes were associated with antioxidant capacity, gluconeogenesis, cellular oxidative stress, and inflammatory stimulus (169). In another study, supplementation of pathogenic Enterobacteriaceae isolates and lactic acid bacteria *in ovo* (10^2^ cfu, *Citrobacter* spp., and *L. salivarius* and *Pediococcus* ssp., e18, amnion) were associated with activation and balance function of the innate and adaptive immune systems (lactic acid bacteria) and attenuation of processes related to the development of the immune system and dysregulation of immunological mechanisms (Enterobacteriaceae) at hatch (170). Besides the early post-hatch effects of probiotic inoculation on proteomic, Rodrigues et al. (171) have shown activation of inflammation pathway in chicks inoculated with lactic acid bacteria or *Citrobacter freundii* (e18, 10^2^ cfu, into amnion) 10 days post-hatch in ileum. Moreover, *in ovo* delivery of lactic acid bacteria was associated with the activation and trafficking of immune cells and skeletal growth. Exposure to Enterobacteriaceae (*C. freundii*) was related to the inhibition of biological function associated with immune cell migration and the inflammatory response (171).

## Pathogen exclusion

The defense mechanism of newly hatched chicks is minimally developed and limited to the innate immune system and maternal antibodies (44). During early post-hatch development, chicks are exposed to many organisms, and due to a low level of colonization of their GIT, they are susceptible to pathogen colonization (44, 104, 172, 173). There are a few mechanisms of competitive exclusion proposed, and they involve the creation of an unfavorable environment for invading bacteria, utilization of receptor sites by commensal bacteria, production of antimicrobial substances, and competition for nutrients allowing for the selection of certain strains of bacteria (174–176). In chicken, administration of 3.5% threonine into the amniotic fluid on e17.5 reduced *Salmonella enteritidis* colonization and ameliorated the negative effects of *Salmonella* infection, including intestinal morphology and integrity, and performance (44). The proposed mechanism of action of threonine involves increased expression of MUC2 gene coding for intestinal mucin and level of mucosal antibodies IgA, and accelerated maturation of the GIT (44). Another study has shown that administration of Bi_2_tos (3.5 mg/egg) on e12 resulted in reduced negative effects of natural *Eimeria* infections, including intestinal lesions and oocyte excretion in Kuroiler chickens (177). Arreguin-Nava et al. (110) showed that injection of defined lactic acid bacteria (10^7^ cfu/200 μl) isolated from adult hens on e19 decreased the Enterobacteriaceae colonization after hatch in chickens challenged with virulent *E. coli* in horizontal infection model. Reduction in Enterobacteriaceae colonization was accompanied by improved body weight gain, reduced mortality at day 7 post-hatch, and variation in cecal microbiota (110).

## Heat stress response

Heat stress negatively affects poultry welfare and productivity (178). Severe physiological changes caused by heat stress include impaired thermotolerance, acid-base imbalance, oxidative stress, and reduced immunocompetence. Behavioral and physiological changes used by chickens to adapt to elevated temperature result in increased mortality, reduced feed intake and body weight gain, and reduced meat quality (179). Management (cooling systems) and nutritional (high-fat diet, supplementation of osmolytes, vitamins, and minerals) strategies have been implemented to mitigate these negative impacts of heat stress on poultry (179). Recent studies have investigated the use of *in ovo* supplementation of bioactive substances to mitigate the negative impacts of heat stress in poultry, especially in broilers due to their fast-growing body and increased metabolic rate (156, 180–183).

The *in ovo* supplementation of L-leucine on e14 and e19 at 25 mm depth into the egg was followed by exposure of the chicks to heat stress at day 9 post-hatch for 3 h (181). The L-leucine supplementation reduced heat shock proteins 70 and 90 in heat-stressed chickens (181). In the same study, heat stress reduced liver arginine and lysine and increased liver glutamine, aspartic acid, and citrulline. Heat stress negatively affected amino acid metabolism; however, the *in ovo* supplementation of L-leucine was not able to mitigate these effects in the heat-stressed chickens (181). Furthermore, the supplementation of 10% γ-aminobutyric acid *in ovo* on e17.5 in the amniotic fluid did not reduce the negative effects of heat stress on various parameters, including rectal temperature, average daily feed intake, average daily gain, and antioxidant balance (180). However, the supplementation of 3.5 mg of Bi_2_tos on e12 downregulated the expression of cytokines IL-4 and IL-12p40. This downregulation could be a result of reduced activation of the immune system. Heat stress is known to damage the intestine and increase the translocation of antigens that can trigger the immune system. It is possible that the downregulation of IL-4 and IL-12p40 is related to intestinal health. Further study needs to clarify this hypothesis (156). In addition, the supplementation of 3.5 mg Bi_2_tos on e12 did not reduce the negative effects of heat stress including breast muscle weight loss, increased feed conversion ratio, and reduced weight gain (182, 183). Although *in ovo* supplementation has shown promise in improving various parameters during the post-hatch period, heat stress is a multifactorial problem and further research is needed to comprehensively define substances that can be used to mitigate the negative consequences of heat stress in chickens.

## *In ovo* effects in other avian species

Most studies related to *in ovo* delivery have been focused on broiler chickens. Information addressing other species is somehow limited in comparison to broilers, but *in ovo* administration of different bioactive substances has been characterized also in other avian species such as turkey, pigeon, quail, and duck.

The *in ovo* supplementation of bioactive substances, including fatty acids, amino acids, and carbohydrates has been investigated in turkeys. The supplementation of branched-chain amino acids (leucine, valine, and isoleucine) increased yolk sac and pancreas weight at hatch and breast muscle weight on e24 and hatch (184). Butyric acid (10, 20, or 30 mg/egg) injected into the yolk sac on e7 improved hatching weight and increased villus height in the duodenum, jejunum, and ileum at day 21 post-hatch. In addition, the feed conversion ratio was reduced, and body weight was increased at hatch and days 21–42 post-hatch (185). Earlier studies have shown that β-hydroxy-β-methyl-butyrate (HMB) and arginine have the potential to improve the development of intestinal digestive and absorptive functions. Foye et al. (186) demonstrated that the interaction between HMB (0 and 0.1%) and arginine (0 and 0.7%) injected on e23 into the amniotic fluid increased the expression of sucrase-isomaltase gene on e25 and those of PEPT-1 and SGLT-1 at hatch. Furthermore, the interaction of HMB and arginine increased jejunal sucrase, maltase, and leucine aminopeptidase activities at e25 and day 14 post-hatch (187). The supplementation of a mixture of dextrin and iodinated casein (75 μg/ml) and dextrin (18% maltodextrin and 10% potato starch dextrin) increased poults' weight at hatch and day 7 post-hatch (188). These results show that *in ovo* supplementation of bioactive substances has the potential to improve intestinal development and function and growth performance post-hatch.

Research on *in ovo* supplementation of bioactive substances in domestic pigeons is limited. The supplementation of 2.5% maltose and 2.5% sucrose on e14.5 in the amniotic fluid increased body weight and pectoral muscle at hatch (189) and showed that *in ovo* supplementation of carbohydrates can enhance intestinal development and digestive functions through increased villus surface area, enhanced brush border enzymes (maltase and sucrase) activities, and upregulation of nutrient transporter genes (SGLT-1 and GLUT-2) (190). Arginine (1%) injected on e13 in the amniotic fluid increased body weight at hatch and day 13 post-hatch, while breast and leg meat yields were increased at day 7 post-hatch (191). Contrary to arginine, histidine supplementation did not affect breast weight (192). In addition, the supplementation of a mixture of amino acids (arginine, lysine, and histidine) at 0.1, 1, or 10% in the amniotic fluid on e13 increased body weight and the relative weight of the heart, kidney, liver, and small intestine at hatch (193). These results suggest that *in ovo* supplementation of amino acids and carbohydrates may help improve embryo development.

Administration of leptin (0.1 or 1 ug per egg) on e5 into the albumen in quail embryos resulted in an earlier hatch and higher post-hatch body weight in treated animals. Moreover, leptin treatment led to changes in endocrine and metabolic parameters such as thyroid hormones and total lipids or triacylglycerol concentrations during post-hatch development (194). Kermanshahi et al. have shown that threonine (5 mg/ml) *in ovo* injection into quail eggs (e11, under the air sac) modulated the MUC2 gene expression post-hatch. At the same time, the injection has no effects on digestive enzyme activity (195). Another study from the same group determined that the lower dose of threonine (5 mg/ml, 50 μl injection volume) increased the mRNA expression level of IgA at hatch (196). The *in ovo* injection of medical plant extracts (1% solution, e5, air cell) such as garlic, ginger, oregano, or cinnamon has been shown to influence the time of sexual maturity and the quality of early eggs laid by Japanese quails (197). The importance of *all-trans* retinoic acid, a metabolite of vitamin A in adipose tissue development has been emphasized by its *in ovo* injection (300 nM) on e9 into quail embryos (198). Additionally, *in ovo* injection of this metabolite increased mRNA expression of adipogenic markers and decreased expression of preadipocyte markers, suggesting the promotion of adipocyte differentiation and decrease population of preadipocytes (198). In another study, Karagecili and Babacanoglu demonstrated that *in ovo* injection of vitamin E and ascorbic acid into the yolk sac or amniotic fluid on e5 of quail eggs did not impact hatchability and quail post-hatch development, but it affected the residual yolk sac absorption, total carotene and concentration of vitamin E derivatives with antioxidant characteristics in the newly hatched quails (199).

In ducks, administration of glutamine, β-hydroxy-β-methylbutyrate, and carbohydrates on e23 into the amniotic fluid has been shown to improve small intestine weight, sucrase and maltase activity post-hatch, and increased pectoral muscle weight at e25 (200, 201). In another study, *in ovo* supplementation of disaccharides and alanyl-glutamine dipeptide on e23 increased plasma glucose concentration post-hatch, liver glycogen content pre-hatch and at hatch, and pectoral glycogen content pre-hatch (202). Additionally, Tangara et al. have shown that *in ovo* carbohydrates and arginine feeding on e23 led to increased body weight of post-hatch ducklings as well as enhanced liver and muscle glycogen storage pre-hatch (203). The *in ovo* administration of IGF-1 (100 ng/embryo, e12, into the albumen) resulted in increased body weight, muscle fiber diameter, muscle cross-sectional area, and the number of myofibers per unit area as well as the number of activated satellite cells and mitotic nuclei in leg and breast muscle of post-hatch ducklings. Moreover, the expression level of MyoD and Myf5 was increased in IGF-1 supplemented ducklings (204). In a second study, Liu et al. have shown that e12 supplementation of IGF-1 into the albumen affected the expression level of myogenic transcription factors such as MyoD and MRF4, muscle fiber parameters, and muscle weight during the late stage of duck embryonic development (205). The same researchers also found that *in ovo* supplementation of follistatin (100 ng/egg, e12) to ducks' embryos affected the expression level of muscle development-related genes. Mainly, Pax7 mRNA expression was upregulated in breast and leg muscle, MyoD mRNA was increased only in leg muscle while Myf5 mRNA was upregulated only in breast muscle. Moreover, the expression of myostatin was downregulated in both muscle types (206). The effects of vitamin C on duck embryos' hatchability were investigated by Nowaczewski et al. (207). They have found that 4 and 8 mg of vitamin C/egg delivered on e20 into the air cell significantly improved the ducks' hatchability by decreasing the number of dead or unhatched embryos (207). Recent data indicate that *in ovo* feeding of vitamin A, L-carnitine, or folic acid (1 mg/egg, e0, air sac) has positive effects on the embryonic development of duck embryos and the health of newly hatched ducklings (208). Primarily, the embryo weight, residual yolk weight, and hatchability percentage were improved in *in ovo* supplemented animals, while body weight, blood parameters, and plasma hormone levels were increased at hatch (208).

## Discussion and conclusion

As characterized in this review, *in ovo* supplementation has been extensively studied in broiler chickens. Regarding other avian species, such as turkey, qual, or duck, the current data are much more limited in comparison to broiler chicken studies. Much more research is needed in those species to obtain the full benefits of this technology. Further research is needed in particular to advance our understanding of *in ovo* technology, particularly the effects of prebiotics and probiotics on intestinal morphological, function, and microbiome development in turkey, duck, quail, and domestic pigeon. Many bioactive substances have been used for injection, with a wide range of physiological responses. The majority of the early studies focused on *in ovo* feeding to supply the embryo with nutrients to facilitate the hatching process and early post-hatch growth, particularly during the time of delayed access to feed. Only in the past few years, there has been an increase in research, focusing on *in ovo* stimulation using mostly prebiotics and synbiotics. Even though it seems that the technology has been researched extensively, many factors still remain inconclusive. For example, the site and timing of injections as well as the volume of injections are not standardized for each bioactive substance, and very often, different methodologies lead to inconclusive or unreproducible results. Therefore, standardization efforts should be made in order to provide a bulletproof methodology for *in ovo* supplementation.

The most efficient way to deliver bioactive substances would be during Marek's disease *in ovo* vaccination. The data on co-delivery are extremely limited. A study by Beck et al. (209) showed no effect of co-delivery of the Marek's disease vaccine (HVT) and probiotics (*Lactobacillus animalis* or *Enterococcus faecium*, e18, amnion, 10^6^ cfu/50 μl volume) on hatchability, performance data, or gastrointestinal parameters (209). The volume of injection as well as the timing and site of delivery need to be taken into consideration for co-delivery. Marek's disease vaccine delivery volume is 50 μl, but for most of the biological substances, much higher volumes are utilized.

Embryo growth, development, and hatchability are affected by many environmental factors and incubation conditions, including egg storage time, incubation temperature, humidity, gas exchange, turning, and light (210, 211). It is clear that any environmental factors compromising embryo development and hatchability will affect the physiological effects of *in ovo* delivery of biological substances. However, we were unable to locate any specific literature addressing the effect of incubation and hatchability conditions together with *in ovo* effects. At the same time, *in ovo* procedure can be one of the factors affecting embryo development and hatching performance (212). However, in most cases, this impact depends on the type of bioactive substances or injection location (213–215). The negative effects are mostly due to the injection process, which leads to a compromised shell and membrane of the egg, allowing for pathogenic bacteria and environmental factors affecting the egg and embryo. The sanitary condition of the injection may lead to compromised eggs and embryos (209). Moghaddam at al. have shown that *in ovo* injection of saline or bioactive substance (royal jelly) significantly decreased hatchability in comparison to the control group (216). Data from de Oliveira et al. (214) suggest only a 10% decrease in hatchability due to inoculation with probiotics. A decrease in hatchability and embryo mortality was also observed by Melo et al. (217). At the same time, there are numerous data indicating no negative *in ovo* effects on hatchability and mortality (115, 218–220), decreased embryo mortality (218), or improved hatchability (221, 222).

Clearly, *in ovo* supplementation with bioactive substances can influence embryonic and post-hatching chick growth, nutrient digestibility, bone development, immune system development, GIT health, GIT microbiota development, heat stress response, and overall health. Even though *in ovo* supplementation has many advantages, the industry use of the method is solely for vaccine delivery right now. There are many reasons why the poultry industry is currently not interested in this methodology, one of which is the lack of a standardized method for *in ovo* feeding and stimulation being one of them. Moreover, more research is needed to show the long-term effects of *in ovo* supplementation and economic advantages for poultry producers. In conclusion, we believe that this method is widely underestimated and underused by the poultry industry. Furthermore, adaptation to commercial settings will require more research efforts and collaborations between researchers and industry partners.

## Author contributions

All authors listed have made a substantial, direct, and intellectual contribution to the work and approved it for publication.
